# A Simplified Working Classification Proposed for Myxoid Tumors of Oral Cavity

**Published:** 2017-04-01

**Authors:** Manas Bajpai, Nilesh Pardhe

**Affiliations:** *Department of Oral and Maxillofacial Pathology, NIMS Dental College, Jaipur, India*

**Dear Editor-in-Chief**

Myxoid tumors of the oral cavity encompass a broad spectrum of lesions, primarily neoplastic (1). Significant variations in the biological behavior ranging from completely harmless to malignant neoplasm require an accurate histopathological diagnosis to ensure a proper treatment (2). A considerable overlapexists between clinical and histopathological features of these group of tumors, which often produces a diagnostic difficulty to clinicians and oral pathologists (3). There is no approved working classification for myxoid tumors of the oral cavity in the literature. A simple working classification of myxoid tumors is proposed here (Table 1)

**Table 1:** A simple working classification for myxoid tumors of oral cavity

**S NO**	**CATEGORY**	**TUMORS**
**1**	Adipose tissue tumors	Myxolipoma Myxiliposarcoma
**2**	Neural tumors	Myxoid neurofibroma Neurothakeoma Malignant peripheral nerve sheath tumor
**3**	Fibroblastic tumors	Myxofibroma Nodular fasciitis Myxofibrosarcoma
**4**	Chondroblastic tumors	Ectomesenchymal chondromyxoid tumor Myxoid chondrosarcoma
**5**	Muscle tumors	Myxoid leiomyosarcoma
**6**	Odontogenic tumors	Odontogenic myxoma
**7**	Miscellaneous	Soft tissue myxoma Ossifying fibromyxoid tumor Malignant ossifying fibromyxoid tumor

This classification is based on the predominance of the areas of myxoid degeneration in histopathology of the tumors of oral cavity. This classification includes adipose, neural, fibroblastic, chondroblastic, muscle, odontogenic and miscellaneous tumors. This classification wouldprovide a better insight into the histopathology of myxoid tumors of oral cavity and wouldbe useful for oral pathologists and cytopathologists.

## References

[1] Graadt van Roggen JF, Hogendoorn PCW, Fletcher CDM (1999). Myxoidtumours of soft tissue. Histopathology.

[2] Za´mecnı´k M (1999). Vascular myxolipoma (angiomyxolipoma) of subcutaneous tissue. Histopathol.

[3] FletcherCDM, UnniKK, Mertens F (2002). World Health Organization Classification of Tumours. Pathology and Genetics of Tumours of Soft Tissue and Bone.

